# INT230-6 shows strong synergy with anti-PD-1 and can induce high complete response rates with T cell memory response in a colon cancer mouse model

**DOI:** 10.1186/2051-1426-3-S2-P352

**Published:** 2015-11-04

**Authors:** Anja C Bloom, Lewis H Bender, Masaki Terabe, Jay A Berzofsky, Ian B Walters

**Affiliations:** 1 National Cancer Institute, Bethesda, MD, USA; 2 Intensity Therapeutics Inc., Westport, CT, USA; 3 NCI/CCR/Vaccine Branch, Bethesda, MD, USA

## 

Intratumorally (IT) administered INT230-6 (a combination of cisplatin, vinblastine and an amphiphilic cell penetration excipient) induces complete responses (CR) in large established tumors, durable protection against re-inoculation, and the ability to shrink and/or resolve non-treated bystander lesions. This therapy results in massive cell death, and recruitment of lymphocytes and DCs into the tumor. Here we explore combinations with checkpoint inhibitors and investigate mechanisms for protection from re-inoculation.

Cohorts of 10 BALB/c mice were inoculated with 1x10^6^ Colon26 cells subcutaneously. Tumors were grown to a mean of 325mm^3^ and then treated with INT230-6 IT daily for 5 days (1 cycle) alone or in combination with checkpoint inhibitors. Control arms included no treatment, and the combination of anti-CTLA4 and anti-PD-1 without drug. Multiple pairwise comparisons were made. INT230-6 x1 was more effective than either control group. Three treatments with INT230-6 tripled the fraction of CRs (from 10 to 30%). Combination with anti-CTLA4 did not add benefit beyond INT230-6 alone. The combination of INT230-6 x1 with anti-PD1 concurrently was more effective than sequentially (50% vs 30% CR and median overall survival (OS) not reached at 99 days vs 44 days). A third (3-way) comparison was adding either anti-PD1 or anti-CTLA4 or both to the INT230-6 x 1 regimen concurrently. INT230-6 with concurrent anti-PD-1 with or without anti-CTLA4 were equivalent and resulted in high CR rates (>50%) and significant prolongation of OS (> 99 days). Both were better than the combination with anti-CTLA4 alone (20% CR and 39-day median OS).

To determine the role of T cell immunity induced by the therapy, all 19 CR animals were re-randomized 102 days after initial treatment to receive either a control IgG, anti-CD4 or anti-CD8 to deplete those T cells, and then re-challenged with the same tumor. Tumors were spontaneously cleared in the IgG group (implying lasting tumor immunity), while all CD8-depleted animals developed new tumors and only 20% of CD4-depleted animals developed tumors, implying that most of the protective immunity was CD8^+^ T cell-mediated.

Together, these results suggest that INT230-6 and anti-PD-1 given concurrently shows significant synergy and can induce durable complete responses in these established colorectal tumor models. The data support the hypothesis that the mechanism of action of this therapy includes induction of tumor specific memory (most importantly CD8^+^) T cells that can address any regrowth or distant metastases (the latter based on rejection of contralateral tumors in our previous studies).

